# Exploiting Three-Dimensional Gaze Tracking for Action Recognition During Bimanual Manipulation to Enhance Human–Robot Collaboration

**DOI:** 10.3389/frobt.2018.00025

**Published:** 2018-04-04

**Authors:** Alireza Haji Fathaliyan, Xiaoyu Wang, Veronica J. Santos

**Affiliations:** Biomechatronics Laboratory, Mechanical and Aerospace Engineering, University of California, Los Angeles, Los Angeles, CA, United States

**Keywords:** action recognition, bimanual manipulation, eye tracking, gaze fixation, gaze object sequence, gaze saliency map, human–robot collaboration, instrumental activity of daily living

## Abstract

Human–robot collaboration could be advanced by facilitating the intuitive, gaze-based control of robots, and enabling robots to recognize human actions, infer human intent, and plan actions that support human goals. Traditionally, gaze tracking approaches to action recognition have relied upon computer vision-based analyses of two-dimensional egocentric camera videos. The objective of this study was to identify useful features that can be extracted from three-dimensional (3D) gaze behavior and used as inputs to machine learning algorithms for human action recognition. We investigated human gaze behavior and gaze–object interactions in 3D during the performance of a bimanual, instrumental activity of daily living: the preparation of a powdered drink. A marker-based motion capture system and binocular eye tracker were used to reconstruct 3D gaze vectors and their intersection with 3D point clouds of objects being manipulated. Statistical analyses of gaze fixation duration and saccade size suggested that some actions (pouring and stirring) may require more visual attention than other actions (reach, pick up, set down, and move). 3D gaze saliency maps, generated with high spatial resolution for six subtasks, appeared to encode action-relevant information. The “gaze object sequence” was used to capture information about the identity of objects in concert with the temporal sequence in which the objects were visually regarded. Dynamic time warping barycentric averaging was used to create a population-based set of characteristic gaze object sequences that accounted for intra- and inter-subject variability. The gaze object sequence was used to demonstrate the feasibility of a simple action recognition algorithm that utilized a dynamic time warping Euclidean distance metric. Averaged over the six subtasks, the action recognition algorithm yielded an accuracy of 96.4%, precision of 89.5%, and recall of 89.2%. This level of performance suggests that the gaze object sequence is a promising feature for action recognition whose impact could be enhanced through the use of sophisticated machine learning classifiers and algorithmic improvements for real-time implementation. Robots capable of robust, real-time recognition of human actions during manipulation tasks could be used to improve quality of life in the home and quality of work in industrial environments.

## Introduction

Recognition of human motion has the potential to greatly impact a number of fields, including assistive robotics, human–robot interaction, and autonomous monitoring systems. In the home, recognition of instrumental activities of daily living (iADLs) could enable an assistive robot to infer human intent and collaborate more seamlessly with humans while also reducing the cognitive burden on the user. A wheelchair-mounted robot with such capabilities could enhance the functional independence of wheelchair users with upper limb impairments (Argall, 2015). During bimanual iADLs, humans rely heavily on vision to proactively gather task-relevant visual information for planning (Johansson et al., 2001). For example, task-relevant information for manipulation could include the three-dimensional (3D) location of an object as well as its structure-related and substance-related properties, such as shape and weight, respectively (Lederman and Klatzky, 1987). Saccades typically precede body movement (Land et al., 1999) and reflect one’s strategy for successful completion of a task.

The relationships between human vision, planning, and intent have inspired roboticists to adopt similar vision-based principles for planning robot movements and to use human gaze tracking for the intuitive control of robot systems. For instance, gaze fixation data collected during the human navigation of rocky terrain have been used to inspire the control of bipedal robots, specifically for the identification and selection of foot placement locations during traversal of rough terrain (Kanoulas and Vona, 2014). Human eye tracking data have also been used in the closed loop control of robotic arms. Recently, Li et al. (2017) demonstrated how 3D gaze tracking could be used to enable individuals with impaired mobility to control a robotic arm in an intuitive manner. Diverging from traditional gaze tracking approaches that leverage two-dimensional (2D) egocentric camera videos, Li et al. presented methods for estimating object location and pose from gaze points reconstructed in 3D. A visuomotor grasping model was trained on gaze locations in 3D along with grasp configurations demonstrated by unimpaired subjects. The model was then used for robot grasp planning driven by human 3D gaze.

In this work, we consider how human eye movements and gaze behavior may encode intent and could be used to inform or control a robotic system for the performance of bimanual tasks. Unlike repetitive, whole-body motions such as walking and running, iADLs can be challenging for autonomous recognition systems for multiple reasons. For instance, human motion associated with iADLs is not always repetitive, often occurs in an unstructured environment, and can be subject to numerous visual occlusions by objects being manipulated as well as parts of the human body. Prior studies on recognition of iADLs often applied computer vision-based approaches to images and videos captured *via* egocentric cameras worn by human subjects. Video preprocessing methods typically consist of first subtracting the foreground and then detecting human hands, regions of visual interest, and objects being manipulated (Yi and Ballard, 2009; Fathi et al., 2011, 2012; Behera et al., 2014; Nguyen et al., 2016).

A variety of methods have been presented for feature extraction for use in machine learning classifiers. In some studies, hand–hand, hand–object, and/or object–object relationships have been leveraged (Yu and Ballard, 2002; Fathi et al., 2011; Behera et al., 2012). The state of an object (e.g., open vs. closed) has been used as a feature of interest (Fathi and Rehg, 2013). Another study leveraged a saliency-based method to estimate gaze position, identify the “gaze object” (the object of visual regard), and recognize an action (Matsuo et al., 2014). Other studies have employed eye trackers in addition to egocentric cameras; researchers have reported significant improvements in action recognition accuracy as a result of the additional gaze point information (Yu and Ballard, 2002; Fathi et al., 2012).

In the literature, the phrase “saliency map” has been used to reference a topographically arranged map that represents visual saliency of a corresponding visual scene (Itti et al., 1998). In this work, we will refer to “gaze saliency maps” as heat maps that represent gaze fixation behaviors. 2D gaze saliency maps have been effectively employed for the study of gaze behavior while viewing and mimicking the grasp of objects on a computer screen (Belardinelli et al., 2015). Belardinelli et al. showed that gaze fixations are distributed across objects during action planning and can be used to anticipate a user’s intent with the object (e.g., opening vs. lifting a teapot). While images of real world objects were presented, subjects were only instructed to mimic actions. In addition, since such 2D gaze saliency maps were constructed from a specific camera perspective, they cannot be easily generalized to other views of the same object. One of the objectives of this work was to construct gaze saliency maps in 3D that could enable gaze behavior analyses from a variety of perspectives. Such 3D gaze saliency maps could be mapped to 3D point clouds trivially obtained using low-cost RGB-D computer vision hardware, as is common in robotics applications. Furthermore, given that all manipulation tasks occur in three dimensions, 3D gaze saliency maps could enable additional insights into action-driven gaze behaviors. Although our experiments were conducted in an artificial lab setting using an uncluttered object scene, the experiment enabled subjects to perform actual physical manipulations of the object as opposed to only imagining or mimicking the manipulations, as in Belardinelli et al. (2015).

The primary objective of this study was to extract and rigorously evaluate a variety of 3D gaze behavior features that could be used for human action recognition to benefit human–robot collaborations. Despite the increasing use of deep learning techniques for end-to-end learning and autonomous feature selection, in this work, we have elected to consider the potential value of independent features that could be used to design action recognition algorithms in the future. In this way, we can consider the physical meaning, computational expense, and value added on a feature-by-feature basis. In Section “Materials and Methods,” we describe the experimental protocol, methods for segmenting actions, analyzing eye tracker data, and constructing 3D gaze vectors and gaze saliency maps. In Section “Results,” we report trends in eye movement characteristics and define the “gaze object sequence.” In Section “Discussion,” we discuss observed gaze behaviors and the potential and practicalities of using gaze saliency maps and gaze object sequences for action recognition. Finally, in Section “Conclusion,” we summarize our contributions and suggest future directions.

## Materials and Methods

### Experimental Protocol

This study was carried out in accordance with the recommendations of the UCLA Institutional Review Board with written informed consent from all subjects. All subjects gave written informed consent in accordance with the Declaration of Helsinki. The protocol was approved by the UCLA Institutional Review Board. A total of 11 subjects (nine males, two females; aged 18–28 years) participated in the study, whose preliminary results were first reported in Haji Fathaliyan et al. (2017). According to a handedness assessment (Zhang, 2012) based on the Edinburgh Handedness Inventory (Oldfield, 1971), two subjects were “pure right handers,” seven subjects were “mixed right handers,” and two subjects were “neutral.”

Subjects were instructed to perform a bimanual tasks involving everyday objects and actions. In this work, we focus on one bimanual task that features numerous objects and subtasks: the preparation of a powdered drink. To investigate how the findings of this study may generalize to other iADL tasks, we plan to apply similar analyses to other bimanual tasks in the future. The objects for the drink preparation task were selected from the benchmark Yale-CMU-Berkeley (YCB) Object Set (Calli et al., 2015b): mug, spoon, pitcher, and pitcher lid. The actions associated with these objects were reach for, pick up, set down, move, stir, scoop, drop, insert, and pour.

**Figure 1:**
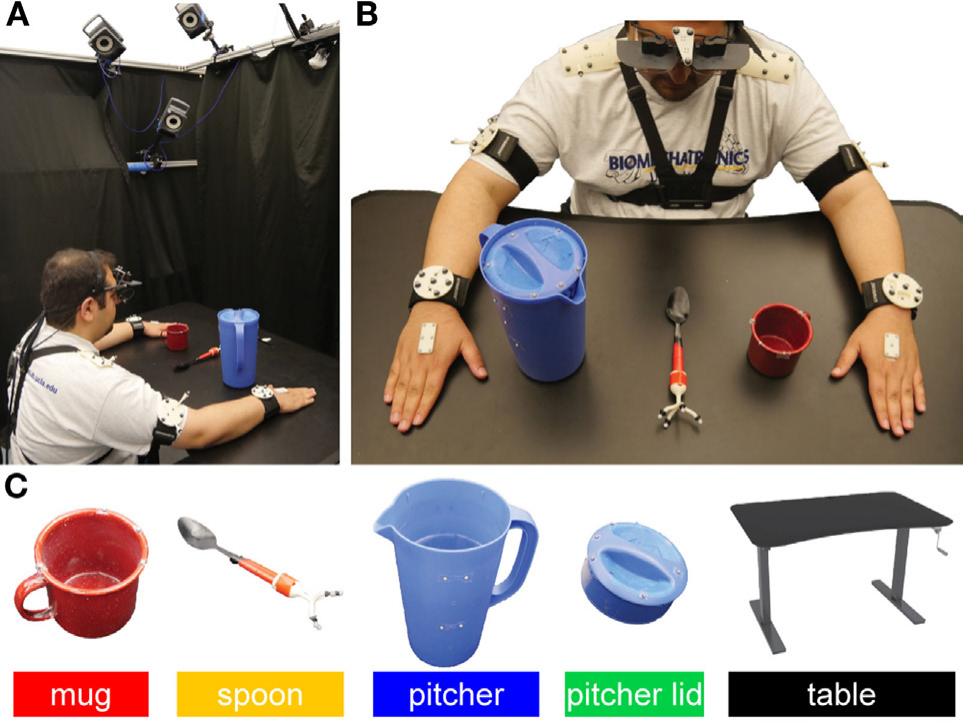
**(A)** Each subject was seated in the motion capture area. A blackout curtain was used to minimize visual distractions. **(B)** The subject wore a head-mounted eye tracker. Motion capture markers were attached to the Yale-CMU-Berkeley objects, the eye tracker, and subjects’ upper limbs. Each trial used the object layout shown. **(C)** Retroreflective markers were placed on a mug, spoon, pitcher, pitcher lid, and table. These objects will be referenced using the indicated color code throughout this manuscript. The subject shown in panels **(A,B)** has approved of the publication of these images.

Subjects were instructed to repeat the task four times with a 1 min break between each trial. The YCB objects were laid out and aligned on a table (adjusted to an ergonomic height for each subject) as shown in Figure 1. The experimental setup was reset prior to each new trial. Subjects were instructed to remove a pitcher lid, stir the contents of the pitcher, which contained water only (the powdered drink was imagined), and transfer the drink from the pitcher to the mug in two different ways. First, three spoonfuls of the drink were to be transferred from the pitcher to the mug using a spoon. Second, the pitcher lid was to be closed to enable to pouring of the drink from the pitcher to the mug until the mug was filled to two-third of its capacity. In order to standardize the instructions provided to subjects, the experimental procedure was demonstrated *via* a prerecorded video.

Subjects wore an ETL-500 binocular, infrared, head-mounted eye tracker (ISCAN, Inc., Woburn, MA, USA) that tracked their visual point of regard, with respect to a head-mounted egocentric scene camera, at a 60 Hz sampling frequency. Calibration data suggest that the accuracy and precision of the eye tracker are approximately 1.43° and 0.11°, respectively. Six T-Series cameras sampled at 100 Hz and a Basler/Vue video camera (Vicon, Culver City, CA, USA) were used to track the motion of the subjects and YCB objects (Figure 1). Retroreflective markers were attached to the YCB objects, eye tracker, and subjects’ shoulders, upper arms, forearms, and hands (dorsal aspects). Visual distractions were minimized through the use of a blackout curtain that surrounded the subject’s field of view.

### Action Segmentation: Task, Subtask, and Action Unit Hierarchy

Land et al. (1999) reported on gaze fixation during a tea-making task. In that work, a hierarchy of four activity levels was considered: “make the tea” (level 1), “prepare the cups” (level 2), “fill the kettle” (level 3), and “remove the lid” (level 4). Spriggs et al. (2009) reported on a brownie-making task and divided the task into 29 actions, such as “break one egg” and “pour oil in cup.” Adopting a similar approach as these prior works, we defined an action hierarchy using a task–subtask–action unit format (Table 1). Subtasks were defined similar to Land et al.’s “4th level activities” while the action units were defined according to hand and object kinematics. All subjects performed all six subtasks listed in Table 1, but not all subjects performed all action units. For example, a couple of subjects did not reach for the pitcher during Subtask 2 (“move spoon into pitcher”).

**Table 1:** Six subtasks (bold) were defined for the task of making a powdered drink; action units were defined for each subtask according to hand and object kinematics.

	Subtask 1: remove pitcher lid	Subtask 2: move spoon into pitcher	Subtask 3: stir inside pitcher	Subtask 4: transfer liquid from pitcher to mug using spoon	Subtask 5: replace pitcher lid	Subtask 6: pour liquid into mug
Action units	Reach for pitcher lid	Reach for pitcher	Stir	Scoop inside pitcher	Reach for pitcher lid	Reach for mug
	Reach for pitcher	Reach for spoon		Reach for mug	Reach for pitcher	Pick up mug
	Pick up pitcher lid	Pick up spoon		Move mug to pitcher	Pick up pitcher lid	Move mug to pitcher
	Set down pitcher lid	Move spoon		Move spoon to mug Drop liquid into mug using spoon Set down mug Set down spoon	Move pitcher lid to pitcher Insert pitcher lid into pitcher	Reach for pitcher handle Pick up pitcher Pour liquid Set down pitcher

**Figure 2:**
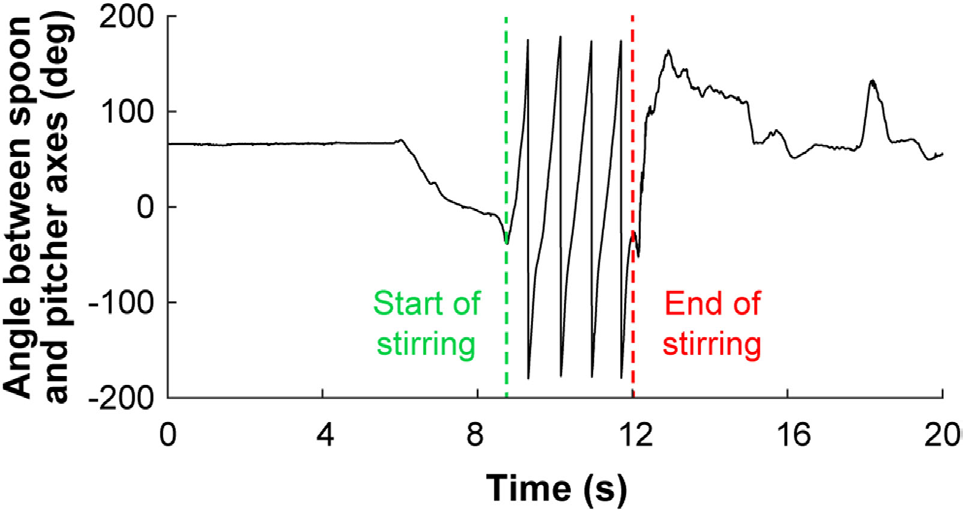
The repetitive nature of the spoon’s kinematics with respect to the pitcher was used to identify the start and end of the action unit “stir inside pitcher.” Although the spoon was not manipulated until approximately 6 s had elapsed in the representative trial shown, the full trial is provided for completeness.

The start and end time of each action unit were identified according to hand and object kinematics and were verified by observing the egocentric video recorded from the eye tracker. For example, the angle of the spoon’s long axis with respect to the pitcher’s long axis and the repetitive pattern of the angle were used to identify the beginning and end of the action unit “stir inside pitcher” (Figure 2).

### Gaze Fixation and Saccade Labeling

**Figure 3:**
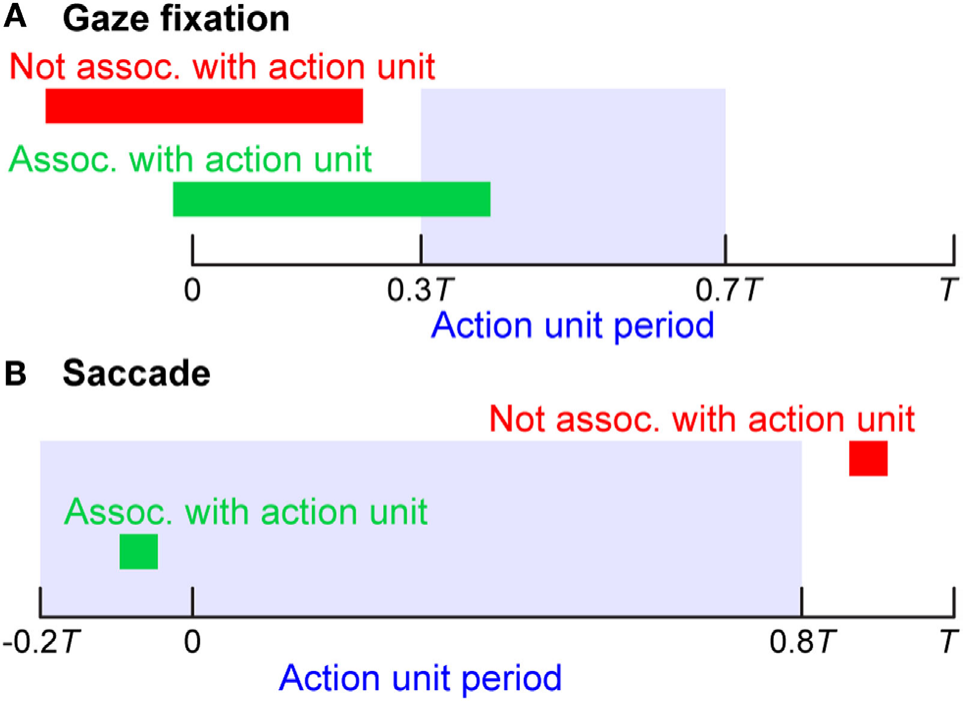
**(A)** A given gaze fixation period was associated with a specific action unit if the gaze fixation period overlapped with the action unit period ranging from 0.3 to 0.7 *T* (blue shaded region), where *T* was the duration of the specific action unit. **(B)** A given saccade was associated with a specific action unit if the saccade occurred during the action unit period ranging from −0.2 to 0.8 *T*.

Saccadic movements of the eye were discovered by Edwin Landott in 1890 while studying eye movements during reading (Kandel et al., 2000). According to Kandel et al., saccadic eye movements are characterized by “jerky movements followed by a short pause” or “rapid movements between fixation points.” In our study, saccades were detected using the angular velocity of the reconstructed gaze vector (see 3D Gaze Vector and Gaze Saliency Map Construction) and intervals between saccades that exceeded 200 ms were labeled as gaze fixations, as in Nyström and Holmqvist (2010). As described previously, the beginning and end of action units were defined based on hand and object kinematics. A heuristic approach, as outlined in Figure 3, was used to associate gaze fixation periods and saccades in the eye tracker data with action units. A given gaze fixation period was associated with a specific action unit if the gaze fixation period overlapped with the action unit period ranging from 0.3 to 0.7 *T*, where *T* was the duration of the specific action unit. A given saccade was associated with a specific action unit if the saccade occurred during the action unit period ranging from −0.2 to 0.8 *T*. Saccade to action unit associations were allowed prior to the start of the action unit (defined from hand and object kinematics) based on reports in the literature that saccades typically precede related motions of the hand (Land et al., 1999; Johansson et al., 2001). The results of the approach presented in Figure 3 were verified through careful comparison with egocentric scene camera videos recorded by the eye tracker.

### 3D Gaze Vector and Gaze Saliency Map Construction

The eye tracker provided the 2D pixel coordinates of the gaze point with respect to the image plane of the egocentric scene camera. The MATLAB Camera Calibration Toolbox (Bouguet, 2015; The MathWorks, 2017) and a four-step calibration procedure were used to estimate the camera’s intrinsic and extrinsic parameters. These parameters enabled the calculation of the pose of the 2D image plane in the 3D global reference frame. The origin of the camera frame was located using motion capture markers attached to the eye tracker. The 3D gaze vector was reconstructed by connecting the origin of the camera frame with the gaze point’s perspective projection onto the image plane.

Using the reconstructed 3D gaze vector, we created 3D gaze saliency maps by assigning RGB colors to the point clouds obtained from 3D scans of the YCB objects. The point cloud for the mug was obtained from Calli et al. (2015a). The point clouds for the pitcher, pitcher lid, and spoon were scanned with a structured-light 3D scanner (Structure Sensor, Occipital, Inc., CA, USA) and custom turntable apparatus. This was necessary because the YCB point cloud database only provides point clouds for the pitcher lid assembly and because the proximal end of the spoon was modified for the application of motion capture markers (Figure 1C). Colors were assigned to points based on the duration of their intersection with the subject’s 3D gaze vector. In order to account for eye tracker uncertainty, colors were assigned to a 5 mm-radius spherical neighborhood of points, with points at the center of the sphere (intersected by the 3D gaze vector) being most intense. Color intensity for points within the sphere decreased linearly as the distance from the center of the sphere increased. Both gaze fixation and saccades were included during RGB color assignment. For each subtask, the RGB color intensity maps were summed across subjects and then normalized to the [0, 1] range, with 0 as black and 1 as red. The normalization was performed with all task-relevant objects considered simultaneously and not on an object-specific basis. This enabled the investigation of the relative visual importance of each object for each subtask.

## Results

### Eye Movements: Gaze Fixation Duration and Saccade Size

**Figure 4:**
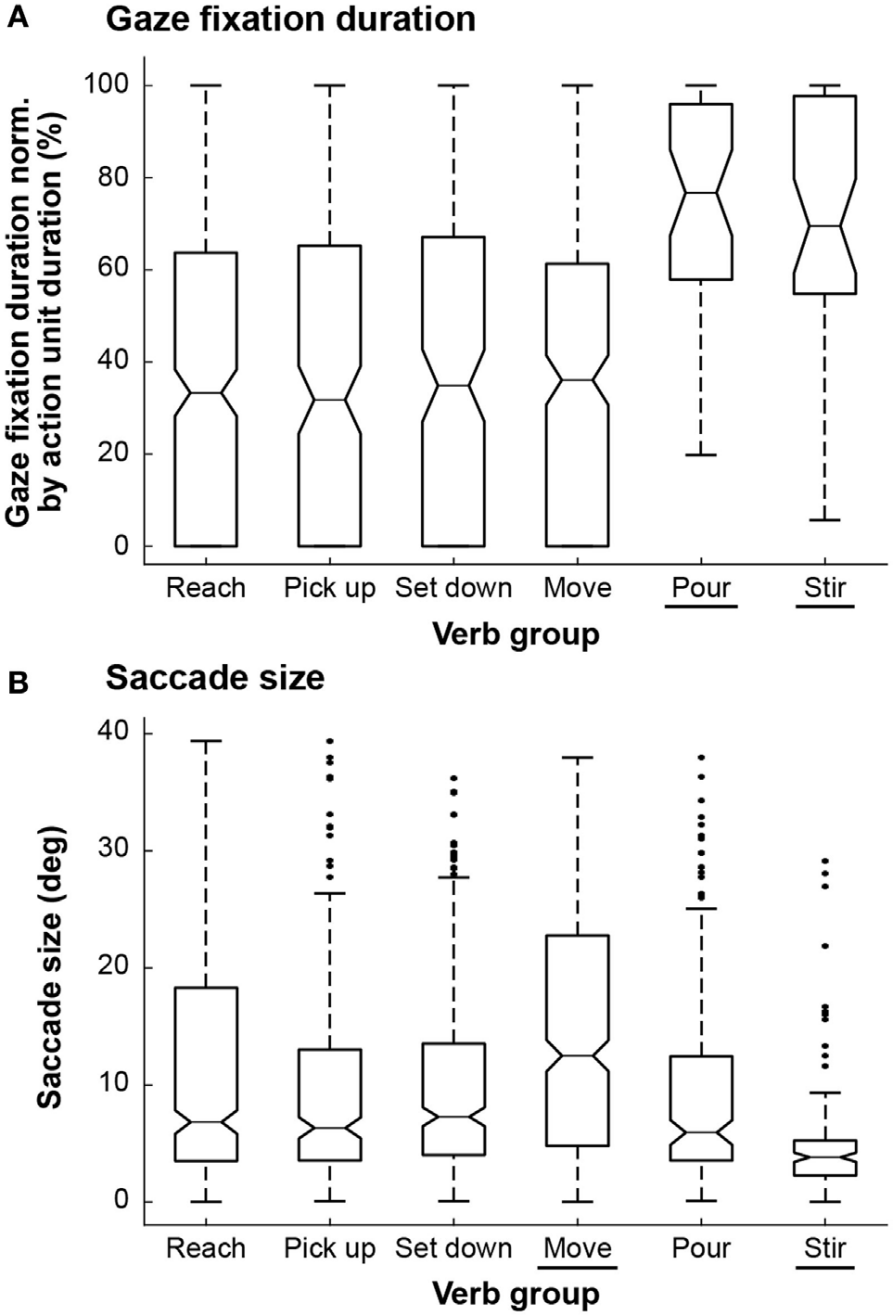
Box and whisker plots are shown for each of the six action unit verb groups for **(A)** normalized gaze fixation duration and **(B)** saccade size. The tapered neck of each box marks the median while the top and bottom edges mark the first and third quantiles. The whiskers extend to the most extreme data points that are not considered outliers (black dots). For normalized gaze fixation duration, both “pour” and “stir” were statistically significantly different from the other action unit verb groups, as indicated by underlines. For saccade size, both “move” and “stir” were statistically significantly different from the other action unit verb groups.

Gaze fixation duration and saccade size have previously been identified as important features for gaze behaviors during iADLs. As in Morrison and Rayner (1981), we use “saccade size” to refer to the angle spanned by a single saccade. Land et al. (1999) reported overall trends and statistics for the entire duration of a tea-making task. However, information about dynamic changes in gaze behavior is difficult to extract and analyze when eye tracker data are convolved over a large period of time. In order to address eye movements at a finer level of detail, we investigated trends in gaze fixation duration and saccade size at the action unit level. Gaze fixation duration data were normalized by summing the durations of gaze fixation periods that belonged to the same action unit and then dividing by the total duration of that action unit. This normalization was performed to minimize the effect of action unit type, such as reaching vs. stirring, on gaze fixation duration results. Gaze fixation duration and saccade size were analyzed according to groupings based on six common action unit verbs: “reach,” “pick up,” “set down,” “move,” “pour,” and “stir” (Figure 4). “Drop” and “insert” were excluded, as they occurred infrequently and their inclusion would have further reduced the power of the statistical tests.

**Figure 5:**
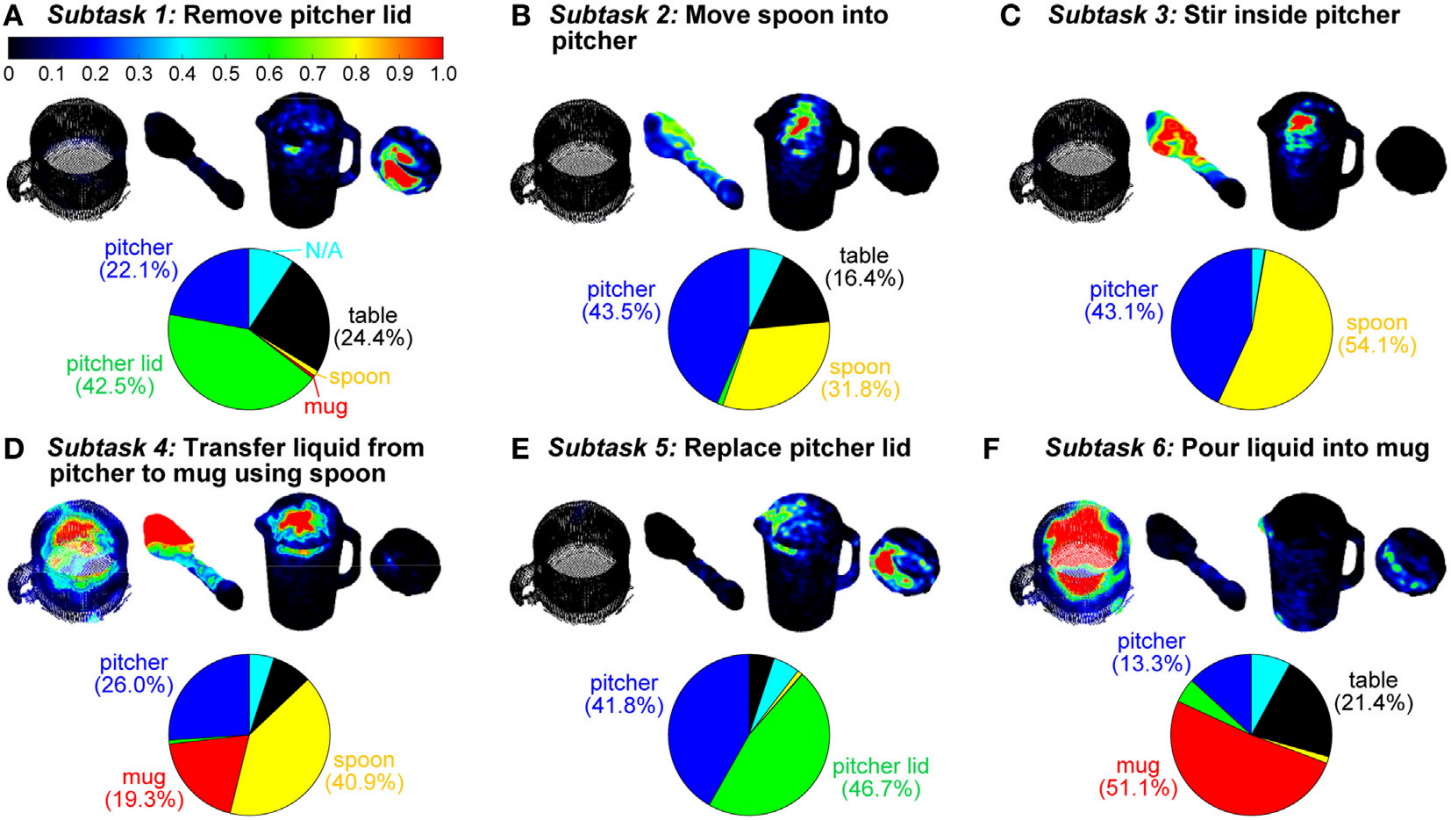
Three-dimensional gaze saliency maps of the task-related objects (mug, spoon, pitcher, and pitcher lid) are shown for each of the six subtasks **(A–F)**. The RGB color maps were summed across subjects and then normalized to the [0, 1] range for each subtask. The RGB color scale for all gaze saliency maps is shown in panel **(A)**. Gaze object percentages are reported *via* pie charts. The colors in the pie charts correspond to the color-coded objects in Figure 1C.

We conducted two ANOVA tests with a significance level of α = 0.05. One test compared the distributions of gaze fixation duration across the six action unit verb groups while the other test compared the distributions of saccade size. In both cases, the ANOVA resulted in *p* < 0.001. Thus, *post hoc* pairwise *t*-tests were conducted to identify which verb groups were significantly different (Table 2). A Bonferroni correction was additionally applied (α = 0.05/*k*, where *k* = 15, the total number of pairwise comparisons) to avoid type I errors when performing the *post hoc* pairwise comparisons. It was found that the average gaze fixation durations for “pour” and “stir” were significantly greater than those of other verbs (Figure 4A). Saccade sizes for “move” and “stir” were significantly different from those of other verbs (Figure 4B). Saccade sizes for “move” were significantly larger than those of other verbs while those for “stir” were significantly smaller (Figure 5).

**Table 2:** The lower left triangle of the table (shaded in gray) summarizes *p*-values for *t*-tests of average normalized gaze fixation duration for different pairs of action unit verbs while the upper right triangle represents *p*-values for *t*-tests with regards to saccade size.

Saccade		Reach	Pick up	Set down	Move	Pour	Stir
Fixation							
Reach			0.012	0.050	3e−6*	0.030	2e−13*
Pick up		0.707		0.450	5e−10*	0.462	3e−12*
Set down		0.242	0.496		3e−10*	0.938	2e−9*
Move		0.666	0.992	0.432		9e−8*	9e−23*
Pour		1e−10*	6e−9*	2e−8*	4e−10*		3e−8*
Stir		3e−9*	1e−7	4e−7*	1e−8*	0.512	

*Asterisks indicate the t-tests that were statistically significant for a Bonferroni-corrected $\alpha =0.00\bar{3}$*.

### 3D Gaze Saliency Maps and Gaze Object Percentages

The 3D gaze saliency map for each object is shown for each of the six subtasks in Figure 5. We use “gaze object” to refer to the object that is intersected by the reconstructed 3D gaze vector. This 3D approach is analogous to the use of 2D egocentric camera videos to identify the gaze object defined as the “object being fixated by eyes” or the “visually attended object” (Yi and Ballard, 2009). In the case that multiple objects were intersected by the same gaze vector, we selected the closest object to the subject as the gaze object. We defined the gaze object percentage as the amount of time, expressed as a percent of a subtask, that an object was intersected by a gaze vector. Gaze object percentages, averaged across all 11 subjects, are presented for each of the six subtasks in pie chart form (Figure 5). Although the table in the experiment setup was never manipulated, during some subtasks, the gaze object percentage for the table exceeded 20% for subtasks that included action units related to “set down.”

### Recognition of Subtasks Based on Gaze Object Sequences

#### The Gaze Object Sequence

**Figure 6:**
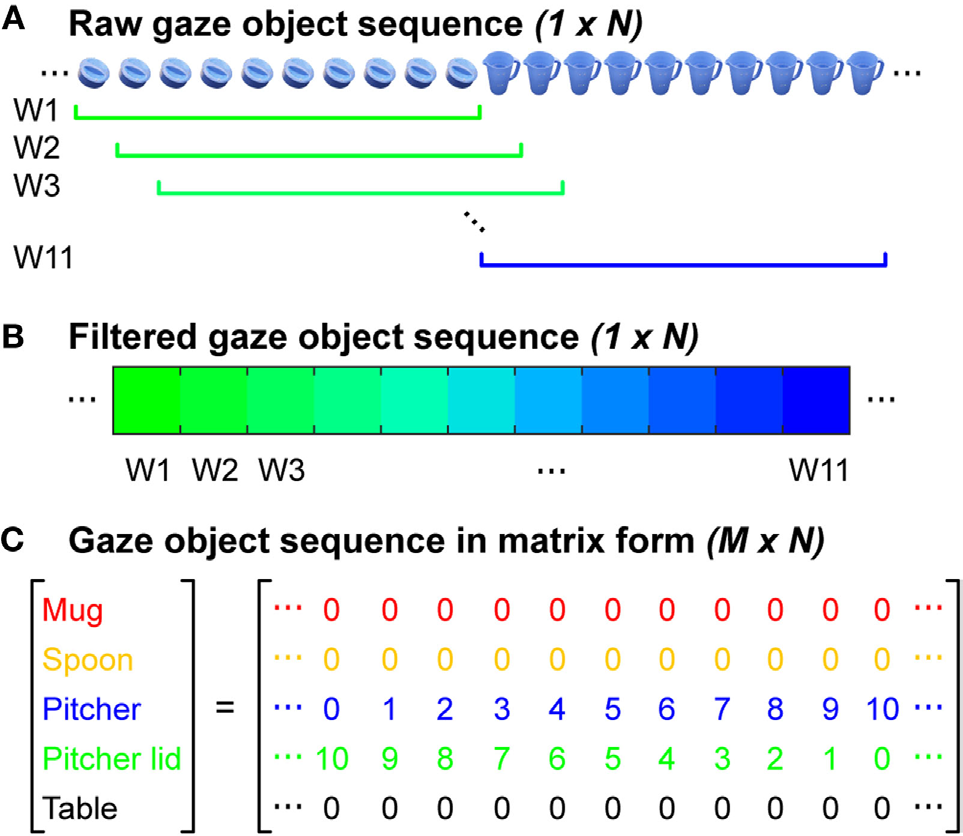
**(A)** Each raw gaze object sequence was represented by a (1 × *N*) set of frames. In this example, the gaze object transitioned from the pitcher lid to the pitcher. The colors in the figure correspond to the color-coded objects in Figure 1C. **(B)** The raw sequence of gaze objects was filtered using a rolling window of 10 frames. **(C)** The gaze object sequence was represented by an (*M* × *N*) matrix for *M* task-relevant objects.

In order to leverage information about the identity of gaze objects in concert with the sequence in which gaze objects were visually regarded, we quantified the gaze object sequence for use in the automated recognition of subtasks. The concept of a gaze object sequence has been implemented previously for human action recognition, but in a different way. Yi and Ballard (2009) performed action recognition with a dynamic Bayesian network having four hidden nodes and four observation nodes. One of the hidden nodes was the true gaze object and one of the observation nodes was the estimated gaze object extracted from 2D egocentric camera videos. In this work, we define the gaze object sequence as being comprised of an (*M* × *N*) matrix, where *M* is the number of objects involved in the manipulation task and *N* is the total number of instances (frames sampled at 60 Hz) that at least one of the *M* objects was visually regarded, whether through gaze fixation or saccade (Figure 6C). Each of the *M* = 5 rows corresponds to a specific object. Each of the *N* columns indicates the number of times each object was visually regarded within a sliding window consisting of 10 frames (Figures 6A,B).

A sliding window was used to filter the raw gaze object sequence to alleviate abrupt changes of values in the matrix. The size of the sliding window was heuristically selected to be large enough to smooth abrupt changes in the object sequence that could be considered as noise, but also small enough so as not to disregard major events within its duration. In preliminary analyses, this sliding window filtration step was observed to improve recognition accuracy.

#### Creating a Library of Characteristic Gaze Object Sequences

Intra- and inter-subject variability necessitate analyses of human subject data that account for variations in movement speed and style. In particular, for pairs of gaze object sequences having different lengths, the data must be optimally time-shifted and stretched prior to comparative analyses. For this task, we used dynamic time warping (DTW), a technique that has been widely used for pattern recognition of human motion, such as gait recognition (Boulgouris et al., 2004) and gesture recognition (Gavrila and Davis, 1995).

Dynamic time warping compares two time-dependent sequences *X* and *Y*, where $X\in {\mathbb{R}}^{S\times U}$ and $Y\in {\mathbb{R}}^{S\times V}$. A warping path $W_{i}=[p_{i1},p_{i2},\ldots ,p_{ij},\ldots ,p_{iK_{i}}]$ defines an alignment between pairs of elements in *X* and *Y* by matching element(s) of *X* to element(s) of *Y*. For example, *p_ij_* = (*u, v*) represents the matched pair of ***x_u_*** and ***y_v_***. If the warping path is optimized to yield the lowest sum of Euclidean distances between the two sequences, the DTW distance between the two sequences *X* and *Y* can be defined as the following:

(1) $$\text{DTW}\left(X,Y\right)=\underset{W_{i}}{\text{min}}\{d\left(W_{i}\right)|W_{i}\in \langle W_{1},W_{2},\ldots ,W_{L}\rangle \},$$

where $d\left(W_{i}\right)=\sum_{j=1}^{K_{i}}\langle p_{ij}\rangle$ and $\langle p_{ij}\rangle =∥x_{u}-y_{v}{∥}_{2}$.

**Figure 7:**
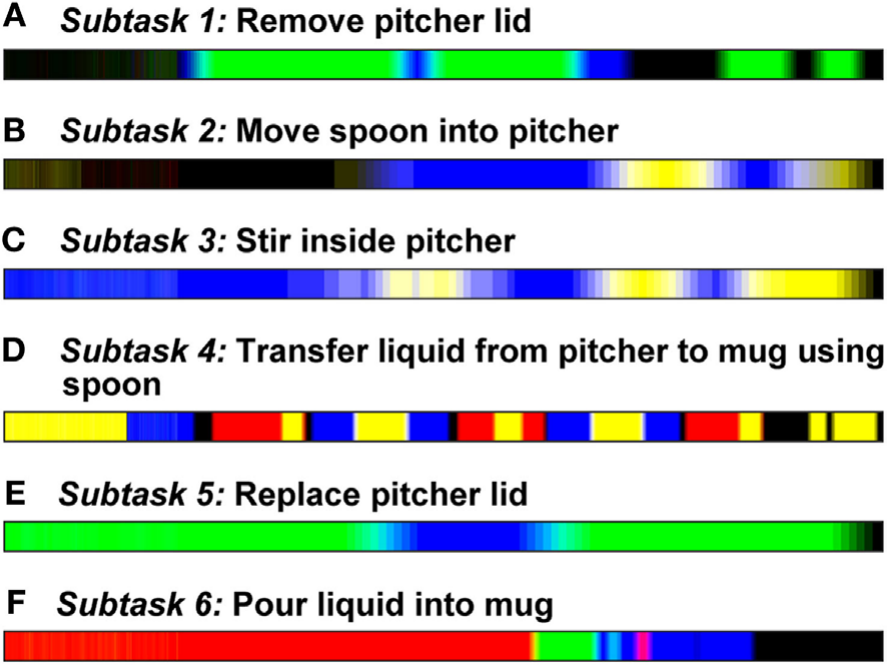
Characteristic gaze object sequences were produced using dynamic time warping barycenter averaging over data from 11 subjects for each of six subtasks **(A–F)**. The colors in the figure correspond to the color-coded objects in Figure 1C. The lengths of the sequences were normalized for visualization.

In order to identify a characteristic gaze object sequence for each subtask, we employed a global averaging method called dynamic time warping barycenter averaging (DBA), which performs the DTW and averaging processes simultaneously. This method uses optimization to iteratively refine a DBA (average) sequence until it yields the smallest DTW Euclidean distance (see Recognition of Subtasks Using DTW Euclidean Distances) with respect to each of the input sequences being averaged (Petitjean et al., 2011). The gaze object sequences were averaged across all trials for all subjects for each subtask using an open source MATLAB function provided by the creators of the DBA process (Petitjean, 2016). A total of 43 trials (4 repetitions per each of 11 subjects, less 1 incomplete trial) were available for each subtask. Figure 7 shows visual representations of the DBA gaze object sequence for each of the six subtasks.

#### Recognition of Subtasks Using DTW Euclidean Distances

Traditionally, the Euclidean distance is used as a metric for similarity between two vectors. However, the Euclidean distance alone is not an accurate measure of similarity for time series data (Petitjean et al., 2011). Here, we use the “DTW Euclidean distance,” which is calculated as the sum of the Euclidean distances between corresponding points of two sequences. The DTW process minimizes the sum of the Euclidean distances, which enables a fair comparison of two sequences. The smaller the DTW Euclidean distance, the greater the similarity between the two sequences. A simple way to associate a novel gaze object sequence with a specific subtask is to first calculate the DTW Euclidean distance between the novel sequence and a characteristic sequence (generated using the DBA process) for each of the six candidate subtasks and to then select the subtask label that results in the smallest DTW Euclidean distance.

**Figure 8:**
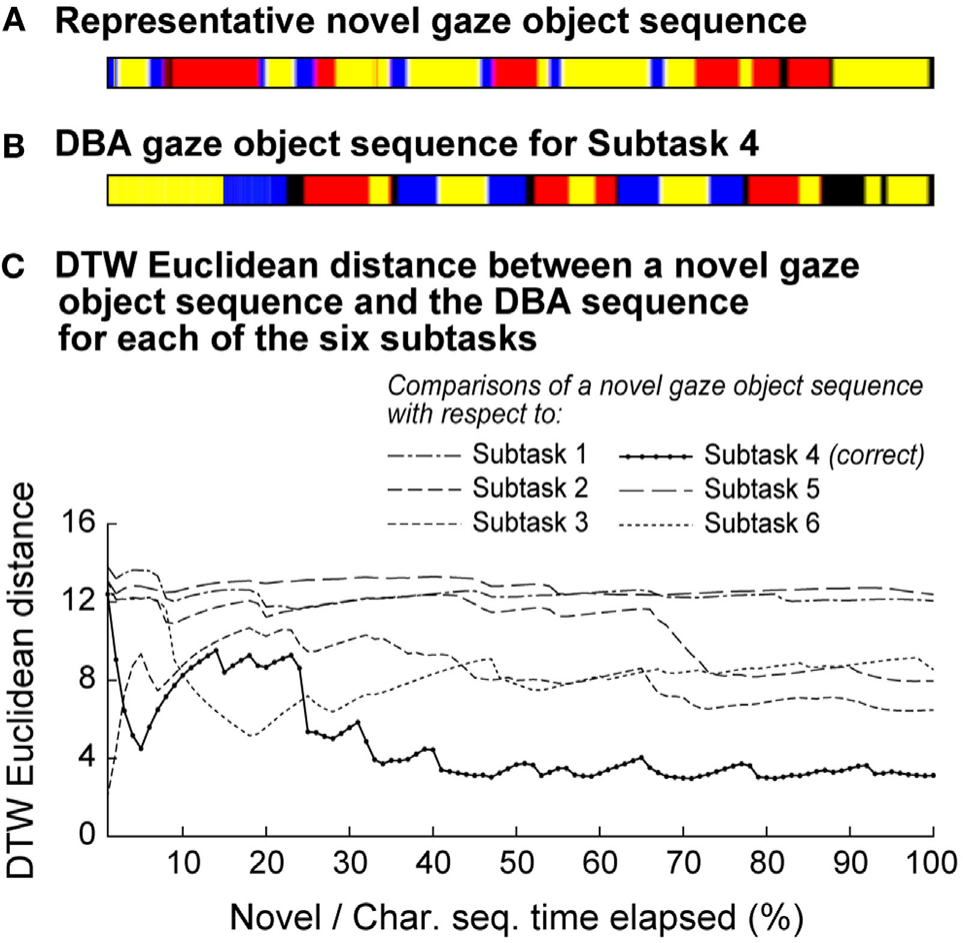
**(A)** A representative novel gaze object sequence is shown. The colors in the figure correspond to the color-coded objects in Figure 1C. **(B)** A DBA gaze object sequence is shown for Subtask 4, which is the correct subtask label for the novel gaze object sequence shown in panel **(A)**. **(C)** The DTW Euclidean distance is shown for the comparisons of a novel gaze object sequence and the DBA sequence for each of the six subtasks. The DTW distance was calculated using equal elapsed times for the novel and DBA sequences. The lowest DTW distance would be used to apply a subtask label. Subtask recognition accuracy generally increases as the elapsed sequence time increases.

Figure 8 shows a novel gaze object sequence and its DTW Euclidean distance with respect to each of the candidate DBA sequences (one for each of six subtasks). The DTW Euclidean distance is reported as a function of the (equal) elapsed times for the novel and DBA gaze object sequences. This enables us to relate recognition accuracy to the percent of a subtask that has elapsed and to comment on the feasibility of real-time action recognition. For instance, for Subtask 4 (“transfer water from pitcher to mug using spoon”), the DTW Euclidean distance between the novel gaze object sequence and the correct candidate DBA sequence does not clearly separate itself from the other five DTW distances until 30% of the novel gaze object sequence has elapsed for the specific case shown (Figure 8). Subtask recognition accuracy generally increases as the elapsed sequence time increases. Figure 8 illustrates how a primitive action recognition approach could be used to label a subtask based on a gaze object sequence alone. However, only one representative novel gaze object sequence was shown as an example.

In order to address the accuracy of the approach as applied to all 43 gaze object sequences, we used a leave-one-out approach. First, one gaze object sequence was treated as an unlabeled, novel sequence. Dynamic time warping barycenter averaging was applied to the remaining sequences. The DTW Euclidean distance was calculated between the novel and candidate DBA sequences, and the pair with the smallest DTW distance was used to label the novel sequence. This process was repeated for each of the gaze object sequences. The DTW distance was calculated using equal elapsed times for the novel and DBA sequences.

**Figure 9:**
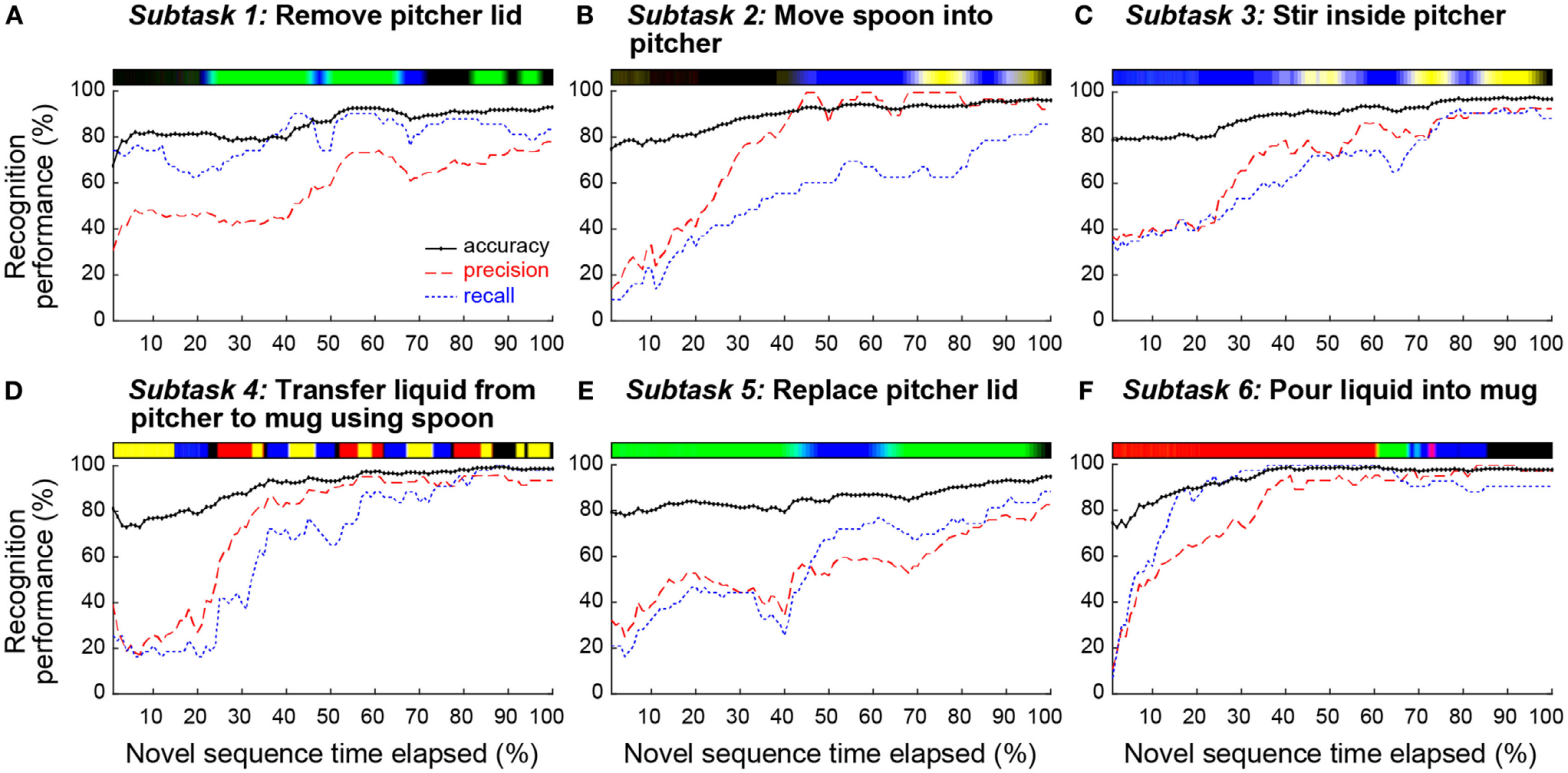
Using a leave-one-out approach, the performance of the action recognition algorithm is reported as a function of the elapsed time of a novel gaze object sequence for each subtask. Accuracy (black solid line), precision (red dashed line), and recall (blue dotted line) are shown for each of the six subtasks **(A–F)**. The characteristic gaze object sequence is shown above each subplot. The colors in the sequence correspond to the objects shown in Figure 1C.

The resulting recognition accuracy, precision, and recall for each subtask are reported in Figure 9 as a function of the percent of the subtask that has elapsed. Accuracy represents the fraction of sequences that are correctly labeled. Precision represents the fraction of identified sequences that are relevant to Subtask *i*. Recall represents the fraction of relevant sequences that are identified (Manning et al., 2008)

(2) $${\text{accuracy}}_{i}=\frac{{\text{TP}}_{i}+{\text{TN}}_{i}}{{\text{TP}}_{i}+{\text{TN}}_{i}+{\text{FP}}_{i}+{\text{FN}}_{i}},$$

(3) $${\text{precision}}_{i}=\frac{{\text{TP}}_{i}}{{\text{TP}}_{i}+{\text{FP}}_{i}},$$

(4) $${\text{recall}}_{i}=\frac{{\text{TP}}_{i}}{{\text{TP}}_{i}+{\text{FN}}_{i}}.$$

TP*_i_*, TN*_i_*, FP*_i_*, and FN*_i_* represent the number of true positive, true negative, false positive, and false negative sequences when attempting to identify all sequences associated with Subtask *i*. For example, consider the task of identifying the 43 sequences relevant to Subtask 1 out of the total of (43*6) unlabeled sequences. Using all sequence data, at 100% elapsed time of a novel gaze object sequence, the classifier correctly labeled 36 of the 43 relevant sequences as Subtask 1, but also labeled 10 of the (43*5) irrelevant sequences as Subtask 1. In this case, TP_1_ = 36, TN_1_ = 205, FP_1_ = 10, and FN_1_ = 7. Using Eqs 2–4, this results in an accuracy of 93.4%, precision of 78.2%, and recall of 83.7% for Subtask 1, as shown in Figure 9A.

**Figure 10:**
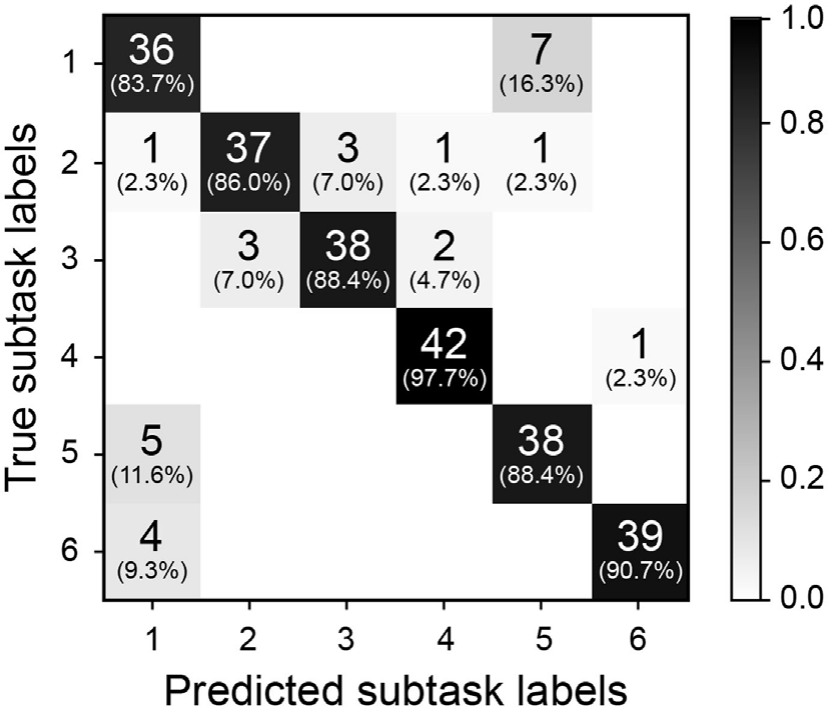
The confusion matrix is shown for 100% of the elapsed time of a novel gaze object sequence for each subtask. Predicted subtask labels (columns) are compared to the true subtask labels (rows). Each subtask has a total of 43 relevant sequences and (43*5) irrelevant sequences. Each shaded box lists the number of label instances and parenthetically lists the percentage of those instances out of 43 relevant subtasks.

Figure 10 shows a confusion matrix that summarizes the subtask labeling performance of our simple action recognition algorithm at 100% of the elapsed time for the novel and DBA gaze object sequences. Predictions of subtask labels (columns) are compared to the true subtask labels (rows). Consider again the task of identifying the 43 sequences relevant to Subtask 1. TP_1_ is shown as the first diagonal element in the confusion matrix (row 1, column 1). FP_1_ and FN_1_ are the sum of off-diagonal elements in the first column and first row, respectively.

## Discussion

### Gaze Fixation Duration and Saccade Size May Reflect Differences in Visual Attention

Eye movements were investigated at the action unit level through gaze fixation duration and saccade size. For gaze fixation duration, both “pour” and “stir” were statistically significantly different from the other action unit verb groups (Figure 4A). The median normalized gaze fixation duration values for “pour” and “stir” were, respectively, 41 and 33% greater than the largest median duration value of the “reach,” “pick up,” “set down,” and “move” verb groups (36% for “move”). The lengthier gaze fixation durations could be due to the fact that pouring and stirring simply took longer than the other movements. The trends could also indicate that more visual attention is required for successful performance of pouring and stirring. For instance, pouring without spilling and stirring without splashing might require greater manipulation accuracy than reaching, picking up, setting down, or moving an object. However, based on the data collected, it is unknown whether subjects were actively processing visual information during these fixation periods. Gaze fixation durations could also be affected by object properties, such as size, geometry, color, novelty, etc. For instance, fixation durations might be longer for objects that are fragile, expensive, or sharp as compared to those for objects that are durable, cheap, or blunt. The effects of object properties on gaze fixation duration and saccade size require further investigation.

For saccade size, both “move” and “stir” were statistically significantly different from the other action unit verb groups (Figure 4B). The relatively large saccade size for “move” was likely a function of the distance by which the manipulated objects were moved during the experimental task. The relatively small saccade size for “stir” (4.7° ± 2.7°) could be due to the small region associated with the act of stirring within a pitcher and the fact that subjects did not follow the cyclic movements of the spoon with their gaze during stirring.

The concept of “quiet eye,” originally introduced in the literature with regards to the cognitive behaviors of elite athletes, has been used to differentiate between expert and novice surgeons (Harvey et al., 2014). Quiet eye has been defined as “the final fixation or tracking gaze that is located on a specific location or object in the visuomotor workspace within 3° of the visual angle for ≥100 ms” (Vickers, 2007). It has been hypothesized that quiet eye is a reflection of a “slowing down” in cognitive planning (not body movement speed) that occurs when additional attention is paid to a challenging task (Moulton et al., 2010). Based on the gaze fixation duration trends (Figure 4A), one might hypothesize that pouring and stirring require additional attention. Yet, “stir” was the only verb group that exhibited a small saccade size in the range reported for quiet eye. We are not suggesting that stirring is a special skill that can only be performed by experts; we would not expect a wide range of skill sets to be exhibited in our subject pool for iADL. Nonetheless, it could be reasoned that certain action units may require more visual attention than others and that gaze fixation and saccade size could assist in recognition of such action units employed during everyday tasks.

### Gaze Saliency Maps Encode Action-Relevant Information at the Subtask and Action Unit Levels

Gaze saliency maps at the subtask level can be used to represent gaze fixation distribution across multiple objects. The gaze saliency maps for the six subtasks (Figure 5) supported Hayhoe and Ballard’s finding that gaze fixation during task completion is rarely directed outside of the objects required for the task (Hayhoe and Ballard, 2005). Considering Subtask 4, (“transfer water from pitcher to mug using spoon”), the objects comprising the majority of the gaze object percentage pie chart (Figure 5D) were grasped and manipulated (spoon) or were directly affected by an action being performed by a manipulated object (pitcher and mug). While the table was not manipulated, it was often affected by action units that required the picking up or setting down of an object, as for the pitcher lid, spoon, and pitcher in Subtasks 1, 2, and 6 (Figures 5A,B,F), respectively. The gaze fixation percentage for the table was dwarfed by the importance of other objects in Subtasks 4 and 5 (Figures 5D,E).

In some cases, a gaze saliency map could be easily associated with a subtask. For instance, gaze saliency was uniquely, simultaneously intense on the spoon bowl and tip, inner wall of the mug, and inner wall of the pitcher for Subtask 4 (“transfer water from pitcher to mug using spoon”) (Figure 5D). In other cases, differences between gaze saliency maps were subtle. For example, the gaze saliency maps were quite similar for the inverse subtasks “remove pitcher lid” and “replace pitcher lid” (Figures 5A,E). In both cases, gaze saliency was focused near the handle of the pitcher lid and the upper rim of the pitcher. However, gaze fixation was slightly more intense near the pitcher spout for Subtask 5 (“replace pitcher lid”) because subjects spent time to carefully align the slots in the pitcher lid with the spout for the “pour liquid into mug” Subtask 6 that was to immediately follow.

**Figure 11:**
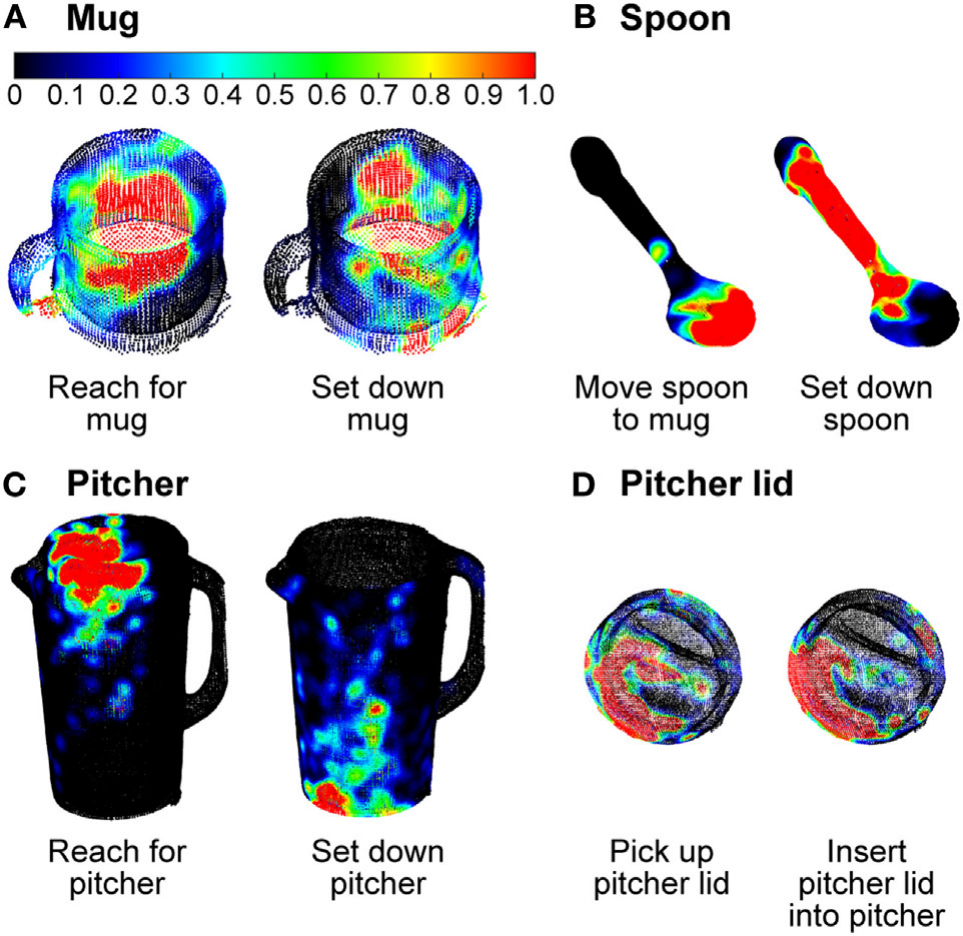
Three-dimensional gaze saliency maps of the task-related objects [mug **(A)**, spoon **(B)**, pitcher **(C)**, and pitcher lid **(D)**] are shown for a subset of action units. The RGB color scale for all gaze saliency maps is shown in panel **(A)**.

Likewise, the gaze saliency maps for Subtask 2 (“move spoon into pitcher”) and Subtask 3 (“stir inside pitcher”) were distinguished only by the subtle difference in gaze fixation distribution on the spoon (Figures 5B,C). The diffuse and homogeneous distribution across the entirety of the spoon for Subtask 2 was contrasted by a focused intensity on the bowl of the spoon for stirring. This was because the “reach for,” “pick up,” and “move” action units performed with the spoon were summed over time to produce the gaze saliency map at the subtask level. Given that the details of each action unit’s unique contribution to the saliency map becomes blurred by temporal summation, it is worth considering gaze saliency maps at a finer temporal resolution, at the action unit level. Due to the short duration of action units (approximately 1 s long), the gaze saliency maps at the action unit level only involve one object at a time. A few representative gaze saliency maps for different action units are shown in Figure 11. The RGB color intensity maps were summed across subjects and then normalized to the [0, 1] range, with 0 as black and 1 as red, according to the duration of the action unit.

Some gaze saliency maps could also be easily associated with specific action units. For instance, gaze saliency intensity was greatest at the top of the pitcher for the action unit “reach for pitcher,” but greatest at the bottom for “set down pitcher” (Figure 11C). By contrast, the gaze saliency maps for the pitcher lid were similar for action units “pick up pitcher lid” and “insert pitcher lid into pitcher.” Subtle differences were observed, such as more focused gaze intensity near the slots in the lid, in preparation for the “pour liquid into mug” Subtask 6 that was to immediately follow. Gaze saliency maps for different action units were also similar for the mug (Figure 11A), possibly due to its aspect ratio. Not only is the mug a relatively small object but also its aspect ratio from the subject’s viewpoint is nearly one. During both “reach for mug” and “set down mug,” gaze fixation was spread around the mug’s centroid. This was surprising, as we had expected increased intensity near the mug’s handle or base for the “reach” and “set down” action units, respectively, based on the findings of Belardinelli et al. (2015). There are a couple of possible explanations for this. First, the Belardinelli et al. study was conducted with a 2D computer display and subjects were instructed to mimic manipulative actions. In this work, subjects physically interacted with and manipulated 3D objects. It is also possible that subjects grasped the mug with varying levels of precision based on task requirements (or lack thereof). For instance, a mug can be held by grasping its handle or its cylindrical body. Had the task involved a hot liquid, for example, perhaps subjects would have grasped and fixated their gaze on the handle of the mug for a longer period.

Although 3D gaze saliency maps are not necessarily unique for all subtasks and action units, it is likely that a combination of the gaze saliency maps for a subtask and its constituent action units could provide additional temporal information that would enable recognition of a subtask. While beyond the scope of this work, we propose that a sequence of gaze saliency maps over time could be used for action recognition. The time series approaches presented for the analysis of gaze object sequences could similarly be applied to gaze saliency map sequences.

#### Practical Considerations and Limitations of Gaze Saliency Maps

If the dynamic tracking of 3D gaze saliency maps is to be practically implemented, one must address the high computational expense associated with tracking, accessing, and analyzing dense 3D point clouds. In this work, the 3D point clouds for the spoon and pitcher were comprised of approximately 3,000 and 20,000 points, respectively. At least two practical modifications could be made to the gaze saliency map representation. First, parametric geometric shapes could be substituted for highly detailed point clouds of rigid objects, especially if fine spatial resolution is not critical for action recognition. The use of a geometric shapes could also enable one to analytically solve for the intersection point(s) between the object and gaze vector. Second, gaze fixation can be tracked for a select subset of regions or segments, such as those associated with “object affordances,” which describe actions that can be taken with an object (Gibson, 1977), or “grasp affordances,” which are defined as “object-gripper relative configurations that lead to successful grasps” (Detry et al., 2009). Computational effort could then be focused on regions that are most likely to be task-relevant, such as the spout, rim, handle, and base of a pitcher. Additionally, techniques can be leveraged from computer-based 3D geometric modeling. For example, triangle meshes and implicit surfaces have been used for real-time rendering of animated characters (Leclercq et al., 2001). A similar approach could be used to simplify the 3D point clouds. In addition to tracking the shape and movement of an object, one could track the homogeneous properties (e.g., RGB color associated with gaze fixation duration) of patch elements of surfaces. The spatial resolution of each gaze saliency map could be tuned according to the task-relevant features of the object and reduced to the minimal needs for reliable action recognition.

One limitation of this work is that we cannot comment on the subject’s true focal point or whether subjects were actively processing visual information. A gaze vector may pass through multiple objects, or even through materials that are not rigid objects (e.g., a stream of flowing water). We calculated the intersection points between a gaze vector and objects in its path and then treated the closest intersection point to the user as a gaze fixation point. This approach may not work if some of the task-relevant objects are transparent and subjects look through one object to visually attend to a more distant object. In this work, objects sometimes passed through the path of a stationary gaze vector, but may not have been the focus of active visual attention. For example, the gaze saliency map for Subtask 3 (“stir inside pitcher”) displayed regions of greater intensity on both the bowl of the spoon and the inner wall of the pitcher (Figure 5C). However, the egocentric camera attached to the eye tracker revealed that the gaze fixation point remained near the water level line in the pitcher. Since the spoon was moved cyclically near the inner wall of the pitcher, in the same region as the surface of the water, the gaze fixation point alternated between the spoon and the pitcher. As a result, both the spoon and pitcher gaze saliency maps were affected. In one case, a subject’s gaze fixation point was calculated as being located on the outer wall of the pitcher during stirring. This interesting case highlights the fact that a direct line of sight (e.g., to the spoon, water, or inner pitcher surface) may not be necessary for subtask completion, and mental imagery (“seeing with the mind’s eye”) may be sufficient (Pearson and Kosslyn, 2013).

Future work should address methods for enhancing the robustness of action recognition algorithms to occlusions. For example, if a gaze object is briefly occluded by a moving object that passes through the subject’s otherwise fixed field of view, an algorithm could be designed to automatically disregard the object as noise to be filtered out. In addition, a more advanced eye tracker and/or calibration process could be leveraged to estimate focal length. Focal length could be combined with 3D gaze vector direction to increase the accuracy of gaze object identification in cases, where the 3D gaze vector intersects multiple objects.

Human gaze behavior “in the wild” will differ to some (as yet unknown) extent as compared to the gaze behavior observed in our laboratory setting. Our use of black curtains and the provision of only task-relevant objects enabled the standardization of the experimental setup across subjects. However, this protocol also unrealistically minimized visual clutter, the presence of novel objects, and distractions to the subject. In a more natural setting, one’s gaze vector could intersect with task-irrelevant objects in the scene. This would result in the injection of noise into the gaze object sequence, for example, and could decrease the speed and/or accuracy of action recognition. Probabilistic modeling of the noise could alleviate this challenge.

### The Gaze Object Sequence Can Be Leveraged for Action Recognition to Advance Human–Robot Collaborations

During everyday activities, eye movements are primarily associated with task-relevant objects (Land and Hayhoe, 2001). Thus, identification of gaze objects can help to establish a context for specific actions. Fathi et al. (2012) showed that knowledge of gaze location significantly improves action recognition. However, action recognition accuracy was limited by errors in the extraction of gaze objects from egocentric camera video data (e.g., failing to detect objects or detecting irrelevant objects in the background), and gaze objects were not treated explicitly as features for action recognition. Moreover, model development for gaze-based action recognition is challenging due to the stochastic nature of gaze behavior (Admoni and Srinivasa, 2016). Using objects tagged with fiducial markers and gaze data from 2D egocentric cameras, Admoni and Srinivasa presented a probabilistic model for the detection of a goal object based on object distance from the center of gaze fixation. In this work, we propose to leverage 3D gaze tracking information about the identity of gaze objects in concert with the temporal sequence in which gaze objects were visually regarded to improve the speed and accuracy of automated action recognition.

In the context of human-robot collaboration, the gaze object sequence could be used as an intuitive, non-verbal control signal by a human operator. Alternatively, the gaze object sequence could be provided passively to a robot assistant that continuously monitors the state of the human operator and intervenes when the human requires assistance. A robot that could infer human intent could enable more seamless physical interactions and collaborations with human operators. For example, a robot assistant in a space shuttle could hand an astronaut a tool during a repair mission, just as a surgical assistant might provide support during a complicated operation. Maeda et al. (2014) introduced a probabilistic framework for collaboration between a semi-autonomous robot and human co-worker. For a box assembly task, the robot decided whether to hold a box or to hand over a screwdriver based on the movements of the human worker. As there were multiple objects involved in the task, the integration of the gaze object sequence into the probabilistic model could potentially improve action recognition accuracy and speed.

The practical demonstration of the usefulness of gaze object sequence is most likely to occur first in a relatively structured environment, such as that of a factory setting. Despite the unpredictability of human behavior, there are consistencies on a manufacturing line that suggest the feasibility of the gaze object sequence approach. The number of parts and tools used during manual manufacturing operations are uniform in their size and shape and are also limited in number. Although the speed with which a task is completed may vary, the task itself is repetitive. Luo et al. (2017) have demonstrated human–robot collaboration for industrial manipulation tasks for which human reaching motions were predicted to enable robot collaboration without collision in a small-shared workspace. In that work, the robot had access to real-time information about the human collaborator’s upper limb kinematics, such as palm and arm joint center positions. Focusing on the safety of human–robot collaboration, Morato et al. (2014) developed a framework that uses a collision avoidance strategy to assist human workers performing an assembly task in close proximity with a robot arm. Numerous RGB-D cameras were used to track the location and configuration of humans within the collaborative workspace. The common theme of such approaches is to track human kinematics and infer intent from kinematic data alone. The additional use of the gaze object sequence could infer human intent at an earlier stage and further advance safety and efficiency for similar types of human–robot collaboration tasks.

The gaze object sequence could also be demonstrated in the familiar environment of someone’s home if a recognition system were properly trained on commonly used objects, where the objects are typically located (e.g., kitchen vs. bathroom), and how they are used. The performance of household robots will largely depend on their ability to recognize and localize objects, especially in complex scenes (Srinivasa et al., 2012). Recognition robustness and latency will be hampered by large quantities of objects, the degree of clutter, and the inclusion of novel objects in the scene. The gaze object sequence could be used to address challenges posed by the presence of numerous objects in the scene. While the combinatorial set of objects and actions could be large, characteristic gaze object sequences for frequently used subject-specific iADLs could be utilized to quickly prune the combinatorial set.

Up to now, we have focused primarily on the task-based aspects of gaze tracking for human–robot collaboration. However, gaze tracking could also provide much needed insight into intangible aspects such as human trust in robot collaborators (Jenkins and Jiang, 2010). Our proposed methods could be used to quantify differences in human gaze behavior with and without robot intervention and could enhance studies on the effects of user familiarity with the robot, human vs. non-human movements, perceived risk of robot failure, etc. Consider, for example, a robot arm that is being used to feed oneself (Argall, 2015). Such a complicated task requires the safe control of a robot near sensitive areas such as the face and mouth and may also be associated with a sense of urgency on the part of the user. A gaze object sequence could reveal high-frequency transitions between task-relevant objects and the robot arm itself, which could indicate a user’s impatience with the robot’s movements or possibly a lack of trust in the robot and concerns about safety. As the human–robot collaboration becomes more seamless and safe, the frequency with which the user visually checks the robot arm may decrease. Thus, action recognition algorithms may need to be tuned to inter-subject variability and adapted to intra-subject variability as the beliefs and capabilities of the human operator change over time.

Other potential applications of the gaze object sequence include training and skill assessment. For instance, Westerfield et al. (2015) developed a framework that combines Augmented Reality with an Intelligent Tutoring System to train novices on computer motherboard assembly. *Via* a head-mounted display, trainees were provided real-time feedback on their performance based on the relative position and orientation of tools and parts during the assembly process. Such a system could be further enhanced by, for example, using an expert’s gaze object sequence to cue trainees *via* augmented reality and draw attention to critical steps in the assembly process or critical regions of interest during an inspection process. Gaze object sequences could also be used to establish a continuum of expertise with which skill level can be quantified and certified. Harvey et al. (2014) described the concepts of “quiet eye” and “slowing down” observed with surgeons performing thyroid lobectomy surgeries. Interestingly, expert surgeons fixated their gaze on the patient’s delicate laryngeal nerve for longer periods than novices when performing “effortful” surgical tasks that required increased attention and cognition. Gaze behavior has also been linked with sight reading expertise in pianists (Truitt et al., 1997). Gaze fixation duration on single-line melodies was shorter for more skilled sight-readers than less skilled sight-readers.

In short, the gaze object sequence generated from 3D gaze tracking data has been demonstrated as a potentially powerful feature for action recognition. By itself, the gaze object sequence captures high-level spatial and temporal gaze behavior information. Moreover, additional features can be generated from the gaze object sequence. For instance, gaze object percentage can be extracted by counting instances of objects in the gaze object sequence. Gaze fixation duration and saccades from one object to another can be extracted from the gaze object sequence. Even saccades to different regions of the same object could potentially be identified if the resolution of the gaze object sequence were made finer through the use of segmented regions of interest for each object (e.g., spout, handle, top, and base of a pitcher).

#### Practical Considerations and Limitations of Gaze Object Sequences

In this work, we have presented a simple proof-of-concept methods for action recognition using a DTW Euclidean distance metric drawn from comparisons between novel and characteristic gaze object sequences. In the current instantiation, novel and characteristic sequences were compared using the same elapsed time (percentage of the entire sequence) (Figure 8). This approach was convenient for a *post hoc* study of recognition accuracy as a function of time elapsed. However, in practice, the novel gaze object sequence will roll out in real-time and we will not know *a priori* what percent of the subtask has elapsed. To address this, we propose the use of parallel threads that calculate the DTW Euclidean distance metric for comparisons of the novel sequence with different portions of each characteristic sequence. For instance, one thread runs a comparison with the first 10% of one characteristic gaze object sequence; another thread runs a comparison for the first 20% of the same characteristic gaze object sequence, etc. Such an approach would also address scenarios in which an individual happens to be performing a subtask faster than the population, whose collective behavior is reflected in each characteristic gaze object sequence. For example, it can be seen that the novel gaze object sequence in Figure 8A has a similar pattern as the characteristic gaze object sequence in Figure 8B. However, the individual subject is initially performing the subtask at a faster rate than the population average. The (yellow, blue, black, red, etc.) pattern occurs within the first 10% of the novel sequence, but does not occur until 30% of the characteristic sequence has elapsed. The delayed recognition of the subtask could be addressed using the multi-thread approach described above Figure 8. To further address the computational expense commonly associated with DTW algorithms, one could implement an “unbounded” version of DTW that improves the method for finding matching sequences, which occur arbitrarily within other sequences (Anguera et al., 2010).

For human-robot collaborations, the earlier that a robot can recognize the intent of the human, the more time the robot will have to plan and correct its actions for safety and efficacy. Thus, practical limitations associated with the computational expense of real-time gaze object sequence recognition must be addressed. At the least, comparisons of a novel sequence unfolding in real-time could be made with a library of characteristic subtask sequences using GPUs and parallel computational threads (one thread for each distinct comparison). The early recognition of a novel subtask is not just advantageous for robot planning and control. The computational expense of DTW increases for longer sequences. Thus, the sooner a novel sequence can be recognized, the less time is spent on calculating the proposed DTW Euclidean metric. Since DTW uses dynamic programming to find the best warping paths, a quadratic computational complexity results. While not implemented in this work, the computational expense of the DTW process could be further reduced by leveraging a generalized time warping technique that temporally aligns multimodal sequences of human motion data while maintaining linear complexity (Zhou and De la Torre, 2012).

#### Potential Advancements for a Gaze Object Sequence-Based Action Recognition System

As expected, recognition accuracy increased as more of the novel gaze object sequence was compared with each characteristic gaze object sequence (Figure 9). However, the simple recognition approach presented here is not perfect. Even when an entire novel gaze object sequence is compared with each characteristic gaze object sequence, the approach only achieves an accuracy of 96.4%, precision of 89.5%, and recall of 89.2% averaged across the six subtasks. The confusion matrix (Figure 10) shows which subtasks were confused with one another even after 100% elapsed time. Although the percentage of incorrect subtask label predictions is low, the subtasks that share the same gaze objects have been confused the most. For instance, the Subtask 1 (“remove pitcher lid”) and Subtask 5 (“replace pitcher lid”) were occasionally confused with one another. It is hypothesized that the training of a sophisticated machine learning classifier could improve the overall accuracy of the recognition results, especially if additional features were provided to the classifier. Potential additional features include quantities extracted from upper limb kinematics and other eye tracker data, such as 3D gaze saliency maps.

As with the processing of any sensor data, there are trade-offs with speed and accuracy in both the spatial and temporal domains. In its current instantiation, the gaze object sequence contains rich temporal information, but at the loss of spatial resolution; entire objects are considered rather than particular regions of objects. By contrast, the 3D gaze saliency map and gaze object percentage contain rich spatial information, but at the loss of temporal resolution due to the convolution of eye tracker data over a lengthy period of time. For practical purposes, we are not suggesting that spatial and temporal resolution should be maximized. In practice, an action recognition system need not be computationally burdened with the processing of individual points in a 3D point cloud or unnecessarily high sampling frequencies. However, one could increase spatial resolution by segmenting objects into affordance-based regions (Montesano and Lopes, 2009), or increase temporal resolution by considering the temporal dynamics of action units rather than subtasks.

While object recognition from 2D egocentric cameras is an important problem, solving this problem was not the focus of the present study. As such, we bypassed challenges of 2D image analysis such as scene segmentation and object recognition, and used a marker-based motion capture system to track each known object in 3D. Data collection was performed in a laboratory setting with expensive eye tracker and motion capture equipment. Nonetheless, the core concepts presented in this work could be applied in non-laboratory settings using low-cost equipment such as consumer-grade eye trackers, Kinect RGB-D cameras, and fiducial markers (e.g., AprilTags and RFID tags).

## Conclusion

The long-term objective of the work is to advance human-robot collaboration by (i) facilitating the intuitive, gaze-based control of robots and (ii) enabling robots to recognize human actions, infer human intent, and plan actions that support human goals. To this end, the objective of this study was to identify useful features that can be extracted from 3D gaze behavior and used as inputs to machine learning algorithms for human action recognition. We investigated human gaze behavior and gaze-object interactions in 3D during the performance of a bimanual, iADL: the preparation of a powdered drink. Gaze fixation duration was statistically significantly larger for some action verbs, suggesting that some actions such as pouring and stirring may require increased visual attention for task completion. 3D gaze saliency maps, generated with high spatial resolution for six subtasks, appeared to encode action-relevant information at the subtask and action unit levels. Dynamic time warping barycentric averaging was used to create a population-based set of characteristic gaze object sequences that accounted for intra- and inter-subject variability. The gaze object sequence was then used to demonstrate the feasibility of a simple action recognition algorithm that utilized a DTW Euclidean distance metric. Action recognition results (96.4% accuracy, 89.5% precision, and 89.2% recall averaged over the six subtasks), suggest that the gaze object sequence is a promising feature for action recognition whose impact could be enhanced through the use of sophisticated machine learning classifiers and algorithmic improvements for real-time implementation. Future work includes the development of a comprehensive action recognition algorithm that simultaneously leverages features from 3D gaze–object interactions, upper limb kinematics, and hand–object spatial relationships. Robots capable of robust, real-time recognition of human actions during manipulation tasks could be used to improve quality of life in the home as well as quality of work in industrial environments.

## Ethics Statement

This study was carried out in accordance with the recommendations of the UCLA Institutional Review Board with written informed consent from all subjects. All subjects gave written informed consent in accordance with the Declaration of Helsinki. The protocol was approved by the UCLA Institutional Review Board.

## Author Contributions

All authors contributed to the conception and design of the study. AF and XW supervised data collection, performed the data analysis, and created the first draft of the figures. All authors wrote sections of the manuscript and contributed to its revision. All authors have read and approved the submitted manuscript.

## Conflict of Interest Statement

The authors declare that the research was conducted in the absence of any commercial or financial relationships that could be construed as a potential conflict of interest.

## Supplementary Material

Video S1 The Supplemental Video shows the experimental setup and reconstruction of 3D gaze saliency maps for the task of preparing a powdered drink.
